# How Does the Social Grouping of Animals in Nature Protect Against Sickness? A Perspective

**DOI:** 10.3389/fnbeh.2021.672097

**Published:** 2021-07-07

**Authors:** Lynette A. Hart, Benjamin L. Hart

**Affiliations:** ^1^Department of Population Health and Reproduction, School of Veterinary Medicine, University of California, Davis, Davis, CA, United States; ^2^Department of Anatomy, Physiology and Cell Biology, School of Veterinary Medicine, University of California, Davis, Davis, CA, United States

**Keywords:** disease exposure, infanticide, infection, pathogen, social behavior

## Abstract

Sickness behavior is broadly represented in vertebrates, usually in association with the fever response in response to acute infections. The reactions to sickness behavior in a group member or potential group member in humans is quite variable, depending upon circumstances. In animals, the reactions to sickness behavior in a group member or potential group member evoke a specific response that reflects the species-specific lifestyle. Groups of animals can employ varied strategies to reduce or address exposure to sickness. Most of these have scarcely been studied in nature from a disease perspective: (1) adjusting exposure to sick conspecifics or contaminated areas; (2) caring for a sick group member; (3) peripheralization and agonistic behaviors to strange non-group conspecifics; and (4) using special strategies at parturition when newborn are healthy but vulnerable. Unexplored in this regard is infanticide, where newborn that are born with very little immunity until they receive antibody-rich colostrum, could be a target of maternal infanticide if they manifest signs of sickness and could be infectious to littermates. The strategies used by different species are highly specific and dependent upon the particular circumstances. What is needed is a more general awareness and consideration of the possibilities that avoiding or adapting to sickness behavior may be driving some social behaviors of animals in nature.

## Introduction

To understand sickness behavior as related to social interactions one should consider animals living in nature – including even ancestral humans – without modern medicine and vaccinations. In social species daily interactions among conspecifics are routine. We assume that behavioral elements of sickness behavior – loss of appetite and thirst, increase in sleep and rest, reduction of movement, transitioning to a heat-conserving posture, and seeking an energy conserving microenvironment – evolved to facilitate the fever response inhibiting the growth of bacterial and viral pathogens until the immune response can take over and expel the pathogen (Hart, 1988, 2011; Hart and Hart, 2019). This ancient fever response, though energetically expensive, has persisted for millions of years, and the highly conserved sickness behavior syndrome occurs in virtually all mammalian, many avian and even some poikilothermic species (Kluger et al., 1996; Hasday et al., 2014).

Among signs of sickness behavior, anorexia seems particularly paradoxical. Febrile animals need calories to fuel the needs of an elevated body temperature and to reduce the demand for muscle breakdown for caloric needs. However, considering animals in nature, one can see that it takes effort to forage or hunt. Both anorexia and a reduced thirst relate to the notion that an animal that does not feel hungry or thirsty has little motivation to move about in search of food and water. An animal staying in one spot engages in much less muscular activity and thus can save on body energy reserves needed for the increased metabolic costs of fever. In response to fever-inducing increase in body temperature suppressing the growth of pathogens, an animal in a febrile state sequesters iron into the liver and spleen. Because iron is important for pathogen growth, by not consuming food the animal reduces the chance of raising blood concentration of iron from foodstuffs (Bullen, 1981; Weinberg, 1984).

The long-term evolutionary survival of this trade-off strategy for consuming food indicates that conserving energy, by staying put and reducing heat loss for a time, is more beneficial than continuing to forage or hunt. Further, a hypothesized functional basis for anorexia is to enhance immune responses (Kyriazakis et al., 1998).

The curled-up position often seen in sick rodents and small canids and felids is more important for heat conservation since the ratio of body surface area to body mass is proportionally greater in small than larger mammals. The body movement involved in grooming results in greater heat loss from more exposure of skin surface and muscular energy expenditure and oral grooming is reduced. Animals that have been sick for several days often have a scruffy, dirty, and oily looking hair coat, which is due to a marked reduction in grooming and letting the removal of ectoparasites by grooming slip by. No oral grooming also minimizes the loss of water through saliva used in licking, which is important for rodents and felids. While a reduction in grooming may lead to more fleas and ticks, the immediate gains in water and energy conservation by ceasing grooming exceed the costs of increasing parasite load. During the recovery phase, the parasite load can be reduced by renewed grooming (Mooring et al., 1996; Eckstein and Hart, 2000).

Referring to sickness behavior as an evolved system with an adaptive value does not imply that survival will usually follow at an individual level. Given that pathogens are pervasive in nature, some increase in the proportion of sick individuals surviving is sufficient to ensure the evolutionary expansion and conservation of the sickness behavior. Sickness behavior is a complex of programmed behaviors that are linked and mostly occur together and are not under conscious control. An animal or human does not generally decide to activate the sickness behavior or inhibit it. Yet, the animal can be motivated to display sickness behaviors by increasing or decreasing their operant response (Holmes and Miller, 1963; Miller, 1964).

The behavior complex or syndrome is linked to fever by being triggered and controlled by the same physiological mechanisms as fever – the increased production of inflammatory cytokines, particularly interleukin-1 (Dantzer, 2001; Kelley et al., 2003). Some new research has focused on sickness behavior being activated without being associated with fever. In some studies, animals have been injected with lipopolysaccharide (LPS), which generally induces sickness behaviors through activation of the immune response (Dantzer et al., 2008). A recent review (Kelley and Kent, 2020) emphasizes the reciprocal communication between the immune system and the brain, and provides examples showing that sickness behavior and fever can be dissociated by social stimuli. Guinea pig pups that were given LPS to induce fever, if the mother were present, had the enhanced fever but not the sickness behavior (Hennessy et al., 2020). A study of febrile and non-febrile children documented the independence of sickness behavior and fever (Corrard et al., 2017). Adults treated with LPS experience symptoms of sickness behavior, without fever, including affective changes like depression (Lasselin et al., 2020a). While the behavioral responses to inflammation-induced sickness have been studied in both rodents and humans, revealing similarities and differences, translational challenges currently preclude making direct comparisons (Lasselin et al., 2020b). Using LPS-induced sickness, extensive early research showed how sickness behavior affects the social behavior of sick animals (e.g., review: Lopes et al., 2012). Vampire bats with induced sickness sharply reduced their social grooming of groupmates, but still were groomed by others (Stockmaier et al., 2018). Thus, despite the programmed nature of sickness behavior, it has plasticity in response to social stimuli (Lopes, 2014). Mating, parental care, early social situations, and agonistic interactions can suppress sickness behavior. Additionally, sickness behavior is modulated by seasonal, sexual, and life-stage effects (Ashley and Wingfield, 2011).

## Brief Review of the General Paradigm of Sickness Behavior, Fever and Response to Pathogens

Humans, their companion animals, farm animals, and wild animals sometimes get sick from a pathogen they contract directly from a conspecific or a substrate such as a water hole. A seriously sick febrile individual living in nature is at a life-or-death juncture. The elements of sickness behavior potentiate the fever response, basically putting all of the animal’s resources into the energy required to raise the febrile thermal set-point and lower the blood levels of iron, suppressing pathogen growth and providing some lead time while the immune system takes over. Specific signs may include diarrhea from intestinal infection or nasal discharge for respiratory infection, but the general elements of the sickness behavior syndrome are the primary signs seen. The physiological linkage between fever and the non-specific signs of depression, inactivity, sleepiness, and anorexia, was established as an evolved, programmed way of having the animal stop looking for food, and moving about, and instead saving vital body resources for fueling the energy-demanding fever response (Hart, 1988).

For social animals, the primary source of pathogens is conspecifics carrying a pathogen to which other conspecifics in the group have no immunity. How do healthy social animals stay well when exposure to sick conspecifics is always a threat? What social dynamics have groups implemented for self-protection from a sick animal? If an animal is actively infected, it will usually show signs of sickness. If it is a member of a group, then group mates may respond in various species-specific ways. If the sick animal is trying to join a group, it will be rejected or peripheralized in various systematic ways. Rather than focusing on aspects of sickness behavior, the remainder of this paper highlights specific strategies that healthy members of social groups pro-actively employ to reduce their likelihood of contracting a disease from a sick conspecific. Some of these strategies have been presented previously from the standpoint of disease avoidance, but without focusing on the social contexts as emphasized here (Hart, 1990).

## Adjusting Exposure to Sick Conspecifics or Contaminated Areas Within a Resident Group

The simple technique of social distancing or withdrawal is a widespread major strategy for disease avoidance throughout the animal kingdom, as documented in recent reviews (Romano et al., 2020; Butler and Behringer, 2021). Using various types of sensory cues to assess risks of infection, invertebrates, fish, amphibians, birds, and mammals all use behavior – social distancing – to avoid pathogens that can be infectious to them. One can assume that most groups of wild animals have members that get sick or injured from time to time. Group members presumably recognize the signs of sickness associated with acute infectious diseases and avoid close contact with the sick one while the sick one goes through the stages of sickness and recovers or dies.

Distancing from contaminated areas is a similar strategy for avoiding infection. A recent study of badgers documents this strategy where hotspots of infection had very few badgers living in those areas, with most badgers living in large groups and specifically avoiding the hotspots (Albery et al., 2020).

For species that are the targets of biting flies that carry a parasite potentially infecting animals, a defense which is the opposite of social separation can be employed. This is crowding in a group to reduce encounters of the flies, a strategy known as the encounter-dilution effect where the animals near the center are the least likely to get bitten (Mooring and Hart, 1992). This is a variant of the selfish herd effect used for avoiding predators where the animals near the center of the group are the least likely to get preyed upon, while those on the periphery are targeted. The animals on the outer edges are the easier targets. A variant of this strategy can be seen in herding species while moving about foraging. If there is a sick group member, it is likely to lag behind the herd as it moves about, and since predators go after what appears to be the most vulnerable group member, this is the one taken. The animal appearing sick is tolerated in the group (presumably at a distance) as bait for predators.

## Caring for an Injured or Sick Group Member

As long known, it is not uncommon for some members of a mammalian group to share food with other group members, such as in felids, canids, hyaenas, and primates (Silk, 1978). Food sharing is particularly prominent in bonobos where they were seen giving up a preferred food to a stranger presumably to facilitate a social interaction (Tan and Hare, 2013). But how do animals respond if a group member is sick? The dwarf mongoose is an outlier in caring for a sick member of the group. As a reflection of the fact that all group members are needed for predator protection, the group provides invalid care for the sick animal, as was found in careful observations of both semi-wild and wild groups (Rasa, 1976, 1983). This means instead of moving from termite hill to termite hill every day or two, they delay the move and stay in one place while the sick group mate recovers (if it does recover).

Mothers of newborn, especially of immature newborn, show highly selected, demanding behavior important to the survival of the offspring. Devoted mothers put their lives at risk to protect their young. If a mother would get sick with a febrile illness, the inactivity and anorexia that generally characterize sickness would interfere with care of the young. Thus, it is interesting that suppression of sickness and fever, even with the occurrence of a pathogen, often occurs at parturition (Harden et al., 2015). Here is an instance where sickness behavior and fever may be diminished with care for the young not hampered by sickness behavior.

## Peripheralization and Agonistic Behaviors to Strange Conspecifics Outside of the Resident Group

For social animals within a group, such as wolves, lions and primates, rejection of non-group conspecifics is an adaptive behavior from several standpoints, including protection of the territory, food resources, females of breeding age, as well as the avoidance of pathogens to which group members may not be immune (Hart, 1990; Hart and Hart, 2018). In nature, group dynamics are often changing, typically with single males moving from one group to another. But the potential male intruder to an established group may well be carrying a pathogen to which the group members are vulnerable, or group members may be sharing a pathogen to which they have immunity but to which the stranger is vulnerable. Strange males are typically peripheralized at first. This is mostly seen in primates, where the strange male may be repeatedly threatened, but with no physical contact. If the stranger comes down with signs of sickness it will not be tolerated, but if remaining normal, let into the group (Altmann and Altmann, 1970; Freeland, 1976).

In one study of dwarf mongooses attempting to join a new group; the strangers trailed the group for an average of 36 days before acceptance, a period of great vulnerability to predation (Rasa, 1987). This type of enforced peripheralization and stress could make the intruder sick if it is harboring a latent infection, and the intruder may not be allowed into the group and may die (Freeland, 1976). At other times the intruder may just hang around, slowly exposing the residents to small doses of foreign pathogens, thus evoking immunity while also acquiring immunity himself to pathogens of the resident group from the small doses he picks up from places such as the water hole. If all goes well, and no signs of sickness emerge, the stranger is integrated into the group.

Another species where there are strict barriers toward intruders, presumably to protect the group from pathogens is the naked mole rat that is blind and relies on olfaction to detect intruders. The territorial animals are aggressive to those of other colonies that lack a distinct colony scent, even excluding colony members that have lost the scent (O’Riain and Jarvis, 1997). When an intruder is detected, a worker sounds an alarm. Naked mole rats also have colony-distinct vocalizations, providing a second major communicative system for identifying colony members, assuring that intruders can be excluded (Barker et al., 2021).

## Using Special Strategies at Parturition When Newborn Are Healthy But Vulnerable

Just after parturition the newborn in many species remain in close contact with the mother. For the newborn having the brain heat up would be detrimental to neural development, and consequently the occurrence of a fever in either the newborn or mother would detrimental. Thus, even with an infection, the occurrence of fever and the associated suite of sickness behaviors, may be suppressed (Harden et al., 2015).

Many females, such as desert bighorn sheep, isolate to private locations prior to parturition, and then some hours after the birth hide their young, visiting the young periodically for feeding, or remaining with young for days or weeks (Karsch et al., 2016). This protective self-quarantine by the healthy female reduces the likelihood of her or her immunocompromised young being exposed to infection from another animal, while also reducing the risk of predation, as with Coke’s hartebeest (Gosling, 1969) and free ranging domestic goats (Lickliter, 1985).

One context that appears not to have been considered for implications of sickness is the infanticide behavior reported in some rodents, canids, and suids (Lukas and Huchard, 2014). Infanticide by females is widespread across mammals especially when females breed in groups and may even involve closely related infants (Lukas and Huchard, 2018). Infanticide (sometimes termed cannibalism in early papers) could protect littermates from an infectious enteric or respiratory disease if one member of the litter is becoming sick and is removed quickly before the pathogen can build up endangering littermates (Hart, 1990). Neonates are mostly resistant to opportunistic pathogens by maternal antibodies in colostrum, but young may occasionally become sick with an environmental pathogen due to insufficient colostrum. A mother reacting to early signs of sickness, such as inactivity and hypothermia, could dispose of the sick infant, saving the littermates from infection. Infanticide can be combined with cannibalism and consuming the dead infant adds to the mother’s nutritional reserves. Veterinarians, laboratory investigators and animal breeders seem to have long recognized that it is the sickly or deformed infants that are likely to be cannibalized (Hart and Hart, 1985; Harkness and Wagner, 1989). There are other explanations for infanticide such as males killing offspring so as to breed the female himself and benefit from the female’s investment in his offspring; this is seen in European wild boars, grizzly bears, Alaskan brown bears, and rodents (Ben-David et al., 2004; Fernández-Llario, 2004; Andersson et al., 2011; Libel et al., 2011; Breedveld et al., 2019). The risk of infanticide can be great enough that it appears to influence choices of nest sites by females seeking to avoid it.

## Conclusion

Our perspective is that sickness behavior plays an important role in animals’ social interactions, as evident when examining animals living in nature. Social behavior of animals, as related to sickness and fever, is important to understand because in nature medical approaches – pharmaceuticals and vaccines – are non-existent. The behaviors remain prominent because they are selected evolutionarily as a result of their control of infectious diseases. Considering sickness behavior and social interactions from the standpoint of disease control is the theme of this paper.

The wide range of social responses to sick animals is very species specific, depending on the species lifestyle. In some groups, especially primates, would-be new members are peripheralized and allowed in the group only after enduring considerable stress from attacks by group members after which they either die from an infection they were carrying or survive. In herding animals, such as antelope, a sick member may be allowed to tag along as “bait” for predators. In a group where all members may be needed for defense against predators, such as in mongoose, considerable time may be invested in caring for a sick one. A wide range of possibilities for social distancing in many different species have been reported (Stockmaier et al., 2021). These different, species-specific reactions related to sickness behavior are not evident in the detailed studies on humans – a single species though very diverse in culture and habitats. In that sense, the studies on humans and animals with regard to sickness behavior and social interactions are complementary.

## Author Contributions

BH and LH: concept, writing, and editing. Both authors contributed to the article and approved the submitted version.

## Conflict of Interest

The authors declare that the research was conducted in the absence of any commercial or financial relationships that could be construed as a potential conflict of interest.
